# Tetrahedral Imidazolate Frameworks with Auxiliary Ligands (TIF-Ax): Synthetic Strategies and Applications

**DOI:** 10.3390/molecules28166031

**Published:** 2023-08-12

**Authors:** Tong Hao, Hui-Zi Li, Fei Wang, Jian Zhang

**Affiliations:** 1 State Key Laboratory of Structural Chemistry, Fujian Institute of Research on the Structure of Matter, Chinese Academy of Sciences, Fuzhou 350002, China; 2 College of Chemistry, Fuzhou University, Fuzhou 350108, China; 3 Fujian College, University of Chinese Academy of Sciences, Fuzhou 350025, China

**Keywords:** zeolite, metal–organic frameworks, zeolitic imidazolate frameworks, tetrahedral imidazolate frameworks, TIF-A1, adsorption and separation

## Abstract

Zeolitic imidazolate frameworks (ZIFs) are an important subclass of metal–organic frameworks (MOFs). Recently, we reported a new kind of MOF, namely tetrahedral imidazolate frameworks with auxiliary ligands (TIF-Ax), by adding linear ligands (Hint) into the zinc–imidazolate system. Introducing linear ligands into the M^2+^-imidazolate system overcomes the limitation of imidazole derivatives. Thanks to the synergistic effect of two different types of ligands, a series of new TIF-Ax with interesting topologies and a special pore environment has been reported, and they have attracted extensive attention in gas adsorption, separation, catalysis, heavy metal ion capture, and so on. In this review, we give a comprehensive overview of TIF-Ax, including their synthesis methods, structural diversity, and multi-field applications. Finally, we also discuss the challenges and perspectives of the rational design and syntheses of new TIF-Ax from the aspects of their composition, solvent, and template. This review provides deep insight into TIF-Ax and a reference for scholars with backgrounds of porous materials, gas separation, and catalysis.

## 1. Introduction

Zeolite and metal–organic frameworks (MOFs) are two kinds of crystalline porous materials [1,2,3]. Both of them have received much attention not only because of their wide applications but also for their fascinating structures that can be rationally designed and predicted [4,5,6,7]. Recently, zeolitic imidazolate frameworks (ZIFs) have been developed by employing tetrahedral metal (M^2+^ = Zn^2+^, Co^2+^, etc.) and imidazolate (im^-^) derivatives to mimic the tetrahedral center and bridge O in zeolite [8,9,10,11,12,13]. At the same time, the M-im-M (M = metal ion) angle is close to the Si-O-Si angle in zeolite. The network diversity of ZIFs is mainly achieved by changing the substitute groups of imidazole or mixing different imidazolate derivatives (link–link interactions) [3,14,15,16,17,18,19,20,21,22]. ZIFs have been widely used in gas adsorption/separation [23,24,25], catalysis [26,27], electrocatalysis [28,29,30,31,32,33,34,35,36,37], biomedicine [38], and more [39,40,41,42,43,44,45,46]. Considering the wide applications of ZIFs, researchers have never stopped exploring and synthesizing new ZIFs with interesting topologies [47,48].

Theoretically, beyond the limitation of imidazole, any bridge ligand with −1 charge can be used to replace imidazole in ZIFs to construct 4-connected frameworks [49]. In fact, the ZIF analogs based on triazole and tetrazole derivatives and the combination of them have also been reported [11,16,50,51,52,53,54,55,56,57]. Additionally, the attempt of some linear ligands generally leads to the formation of the non-zeolite dia (cubic diamond) topology, suggesting the importance of bent ligands.

**Scheme 1:**
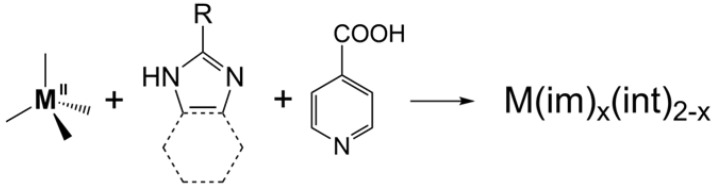
Strategy for the synthesis of TIF-Ax.

In 2011, we reported a new kind of MOF, namely tetrahedral imidazolate frameworks with auxiliary ligands (TIF-Ax), by adding linear ligands into the zinc–imidazolate system (Scheme 1) [22]. The most obvious difference between TIF-Ax and ZIFs is that the introduction of Hint makes the angle of M-int-M larger than that of M-im-M. Therefore, introducing linear ligands into the M^2+^-imidazolate system overcomes the limitation of imidazole derivatives. Thanks to the synergistic effect of two different types of ligands, a series of new TIF-Ax with interesting topologies and a special pore environment has been reported, and they have attracted extensive attention in gas adsorption, separation, catalysis, heavy metal ion capture, framework flexibility, and so on. More importantly, a variety of facile and rapid synthesis methods have been developed to realize the near-kilogram synthesis of them, paving the way for the large-scale application of TIF-Ax in the future.

As a typical example of a ZIF-like compound, the structure of TIF-A1 has been introduced in several reviews [3,26,58,59,60,61]. In this review, we give a comprehensive overview of TIF-Ax, including their synthesis methods, structural diversity, and multi-field applications. We list all the reported structures (Table 1) and summarize the synthesis methods of TIF-Ax, including the solvothermal method, reflux method, microwave-assisted method, and solvent-free method. In addition, their current multi-field applications are determined and summarized. Finally, we also discuss the challenges and perspectives of the rational design and syntheses of new TIF-Ax from the aspects of their composition, solvent, and template. This review provides deep insight into TIF-Ax and a new impetus for their development.

## 2. Synthesis Method

All of the TIF-Ax compounds were initially synthesized using the solvothermal method (for detailed information, refer to the original papers). Based on the solvothermal method, diverse improved methods have been developed to serve various purposes.

### 2.1. Facile Synthesis

TIF-A1 was originally synthesized by the solvothermal method using Zn(NO_3_)_2_∙6H_2_O, adenine, and isonictinic acid (Hint) in *N*,*N*′-dimethylformamide (DMF) at 120 °C for 3 days with a high yield (88%) [22].

#### 2.1.1. Room Temperature Synthesis

The first choice for simple synthesis is room temperature synthesis, which avoids the use of external energy. For example, using the same reactants as the original method, highly crystalline TIF-A1 was produced by allowing the reaction to proceed at room temperature for 3 days. However, the yield in this case was low (14.7%) [67].

#### 2.1.2. Ultrasonic and Stirring Synthesis

On the basis of the room temperature method, ultrasonic and stirring techniques were introduced to improve the yield within a shorter time. Ultrasonic and stirring processes facilitate thorough mixing of the reactants, promoting uniform nucleation during the reaction, and thus, achieving the goal of rapid synthesis. The experimental results show that the yields of TIF-A1 were improved with ultrasonic and stirring (31.1% and 30.7%, respectively) within 2.5 h [67]. Furthermore, the yield of TIF-A1 was further enhanced through the combination of stirring and heating at 120 °C for only 30 min (reaching 83%), a value that closely approached the maximum achievable yield of 88% [67].

### 2.2. Metal Sources

Inspired by the aforementioned outcomes achieved through stirring and heating at 120 °C in a brief timeframe, and mindful of the potential for the reaction between nitrate and carboxylic acid to generate corrosive nitric acid, which might compromise the equipment’s integrity and influence the stability of the final MOFs, alternative metal sources were employed to investigate the feasibility of substituting Zn(NO_3_)_2_∙6H_2_O. Similarly, the yields of TIF-A1 synthesized from ZnO and Zn(CH_3_COO)_2_∙2H_2_O were 75.5% and 80.5%, respectively [67]. This shows that the metal source has good substitutability.

### 2.3. Upscale Synthesis

**Figure 1:**
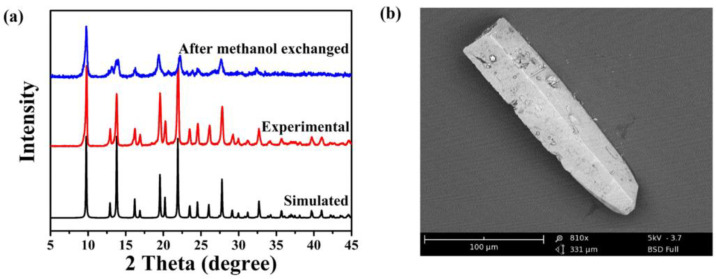
Characterization of upscale synthesis TIF-A1: (**a**) powder XRD patterns; (**b**) scanning electron microscopy (SEM) images. Adopted permission from Ref. [67]. Copyright © 2023, Elsevier Inc.

The potential for synthesizing a kilogram of TIF-A1 was further investigated using the aforementioned methods. Interestingly, through the scaling up of reactant quantities, a yield of over 800 g of TIF-A1 was achieved in a single heating process at 120 °C [67]. The resulting samples exhibited a similar crystallinity, morphology, and BET surface area to those obtained through the original solvothermal method (as depicted in Figure 1 and summarized in Table 2).

## 3. Structure Diversity of TIF-Ax

TIF-A1 is a 4-connected metal–organic framework with Zn^2+^ as the metal center, adenine (ad) as the main ligand, and isonicotinic acid (Hint) as the auxiliary ligand. Both ligands bind to Zn^2+^ via N and carboxyl O on heterocycles. It was observed that TIF-A1~A3 has a different topology, which was regulated by changing the type of imidazole salts in the framework. 2-NH_2_-TIF-A1 and 3-NH_2_-TIF-A1 were prepared by functional modification, and their functional groups could effectively adjust the pore environment and pore size. Cd-ad-int (int= isonicotinic acid) changed the metal center to have different building units, thus adjusting the structural network. Zn-thp-nit (thp = theophylline, nit = nicotinic acid) changed the isonicotinic acid ligand into nicotinic acid, which changed the angle of connection between the ligand and the metal. In TIF-A4 ~ A8, monocarboxylic acid with a coordination angle of ca. 120° was selected to replace the Hint, and a dia topological structure and layered structure were obtained. The series structures of TIF-Ax are briefly described below.

### 3.1. TIF-A1~A3

**Figure 2:**
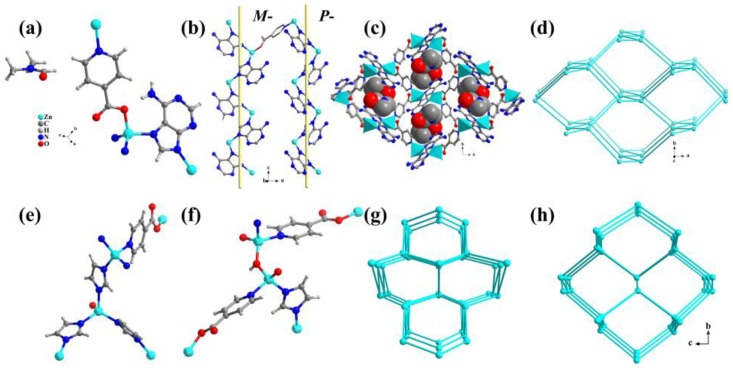
(**a**) The molecular structure of TIF-A1. (**b**) The *P*-helix and *M-*helix in TIF-A1. (**c**) The 3D framework of TIF-A1 with guest DMF molecules (space-filling mode) in the channels. (**d**) The dmp topology of TIF-A1. (**e**) The molecular structure of TIF-A2. (**f**) The molecular structure of TIF-A3. (**g**) The dia topology of TIF-A2. (**h**) The neb topology of TIF-A3.

**Scheme 2:**
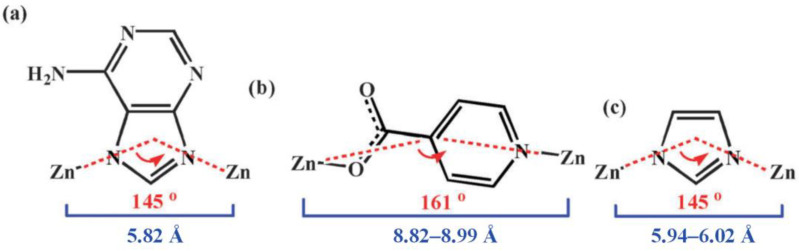
The bond distances and bond angles of the linkers: (**a**) adeninate; (**b**) isonictinate; (**c**) imidazolate. Adopted permission from Ref. [22]. Copyright © 2023, Royal Society of Chemistry.

In 2011, we reported the syntheses of TIF-A1~A3 with different topologies. In TIF-A1, the Zn^2+^ ion is tetrahedrally coordinated by two ad and two int ligands (Figure 2a). The ad ligands link the Zn^2+^ sites using two N donors from its imidazolate ring into infinite 2_1_ helices along the *c*-axis. The ratio of right-handed (*P-*) to left-handed (*M-*) helices is 1:1, and the two helices are connected by the int ligands (Figure 2b). Each int ligand bridges two Zn^2+^ sites through the coordination of one carboxyl O atom and the pyridyl N atom to generate a zigzag chain along the *b*-axis. The use of linear ligands leads to larger Zn-Zn distances linked by int (8.82~8.99 Å) and the Zn-int-Zn angle (ca. 160°). In comparison, the Zn-ad-Zn angle is 145°, and Zn-Zn distances linked by ad are about 5.83 Å, which are similar to other reported ZIFs (Scheme 2). The Zn-ad-Zn helices and Zn-int-Zn chains share Zn^2+^ sites to make the 3D framework (Figure 2c) with dmp topology (Figure 2d) [22]. Dmp topology has never been reported in zeolites and ZIFs, which is mainly attributed to the employment of a linear ligand. The framework of TIF-A1 contains 1D rhombic channels with a size of 8.0 × 7.0 Å along the *c*-axis. These large channels are occupied by the guest DMF.

Each Zn^2+^ of Zn_2_(im)_3_(int) (TIF-A2) is connected by three im (imidazolate) ligands and one int auxiliary ligand to form a dia topological framework (Figure 2e,g) [22]. In TIF-A3, each Zn^2+^ is coordinated by one im ligand, two int ligands, and one unique OH- to form a neb topological framework (Figure 2f,h). The μ_2_-OH linker in TIF-A3 is similar to the μ_2_-O in zeolite. In addition, both TIF-A2 and TIF-A3 exhibit a 2-fold interpenetrating framework.

### 3.2. 2-NH_2_-TIF-A1 and 3-NH_2_-TIF-A1

**Figure 3:**
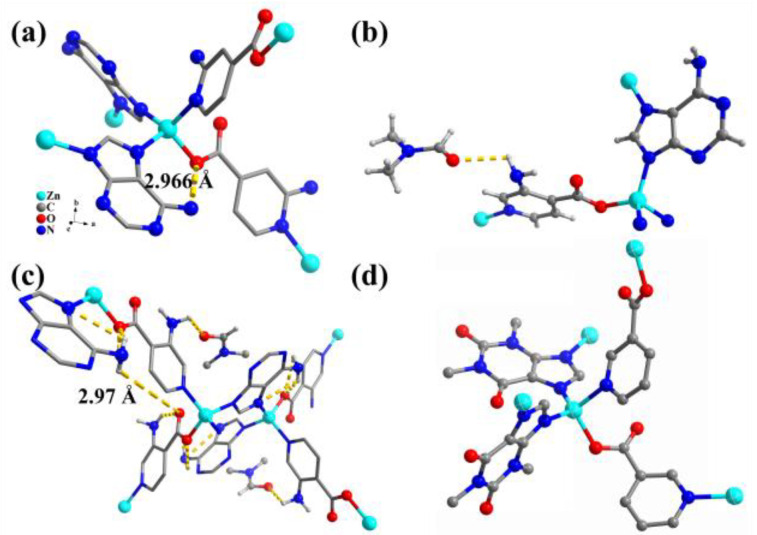
(**a**) The coordination environment and hydrogen bonding interaction (yellow dashed line) in the structure of 2-NH_2_-TIF-A1. (**b**) The coordination environment of [Zn(3-NH_2_-int)(ad)](DMF). (**c**) N-H∙∙∙O and N-H∙∙∙N hydrogen bond networks (dash line) for [Zn(3-NH_2_-int)(ad)](DMF). (**d**) The structural unit in [Zn(thp)(nit)].

Introducing functional groups is a crucial approach to enhancing the performance of MOFs. In 2013, we synthesized 2-NH_2_-TIF-A1 by using 2-aminoisonicotinic acid (2-NH_2_-Hint) instead of Hint. In 2-NH_2_-TIF-A1, the amino group (-NH_2_) of 2-NH_2_-int ligand was oriented toward the interior of the cavity, allowing for adjustments to the pore size and the environment of the resulting 2-NH_2_-TIF-A1 structure. Simultaneously, the formation of an intramolecular hydrogen bond between the amino groups of the ad ligand and the carboxyl oxygen in the int ligand contributed to enhancing the stability of NH_2_-TIF-A1 (Figure 3a) [62]. Similarly, the utilization of 3-aminoisonicotinic acid (3-NH_2_-Hint) as an auxiliary ligand led to the construction of isostructural 3-NH_2_-TIF-A1 (Figure 3b,c) [63]. In 3-NH_2_-TIF-A1, the presence of an intramolecular hydrogen bond between the amino groups of the ad ligand and the carboxyl oxygen in the int ligand was also observed. Furthermore, the -NH_2_ group of the 3-NH_2_-int ligand acted as a bonding site for anchoring guest molecules, such as DMF and DMA.

### 3.3. Zn-thp-nit

In 2013, Lou and co-workers synthesized [Zn(thp)(nit)] 2D layered structures from theophylline (Hthp) and nicotinic acid (Hint) mixed ligands [64]. Each thp was attached to Zn^2+^ by two pyridyl N atoms, while each nit auxiliary ligand was attached to Zn^2+^ by one carboxyl O atom and one pyridyl N atom (Figure 3d). The thp ligand was connected with Zn^2+^ to form a one-dimensional chain, and the polymeric chain was further connected with Zn^2+^ through nit to form a 2D coordination network.

### 3.4. Cd-ad-int

**Figure 4:**
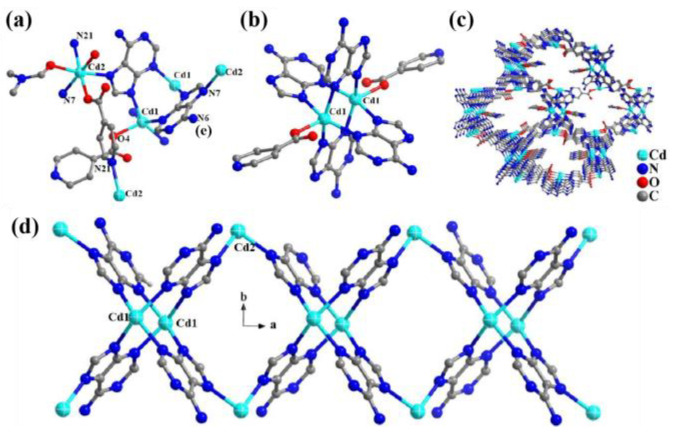
(**a**) The coordination environment of the Cd atoms in Cd-ad-int. (**b**) The cadmium(II)–adenine paddle-wheel subunit. (**c**) View of the 3D framework of Cd-ad-int along the a-axis. (**d**) The cadmium(II)–adenine chain.

In 2012, Cd-ad-int was synthesized by using Cd(NO_3_)_2_∙4H_2_O to replace Zn salt [65]. The structural unit of Cd-ad-int consists of two cadmium atoms (Cd1 and Cd2), two deprotonated adenine (ad) and two int ligands. The Cd1 atom adopts a square pyramidal mode and is penta-coordinated with four N atoms from four different adenines and one oxygen O4 atom from int (Figure 4a). The coordination configuration of Cd2 is octahedral, with six bonded atoms consisting of three oxygen atoms (one water molecule, one DMF molecule, one carboxyl group of int), three nitrogen atoms (N7 and N16 of adenine, and N21 of int). Each adenine holds together three cadmium atoms in the form of a μ3-linker.

Cd-ad-int contains a Cd_2_(ad)_4_ paddle-wheel unit with an axial position occupied by two int ligands (Figure 4b). The Cd_2_(ad)_4_ unit is further bridged by Cd_2_ atoms to form a chain along the a-axis (Figure 4d). The two int ligands have two different functions, one connecting two adjacent Cd_2_ atoms, and the other coordinating with Cd1 atoms as a side-chain ligand. By sharing Cd_2_ atoms, the above two different types of chains are cross-linked to each other to form a three-dimensional skeleton containing a one-dimensional channel (Figure 4c). The free space in the channel is occupied by pendant ligands and coordinating solvent molecules.

### 3.5. TIF-A4~A8

**Figure 5:**
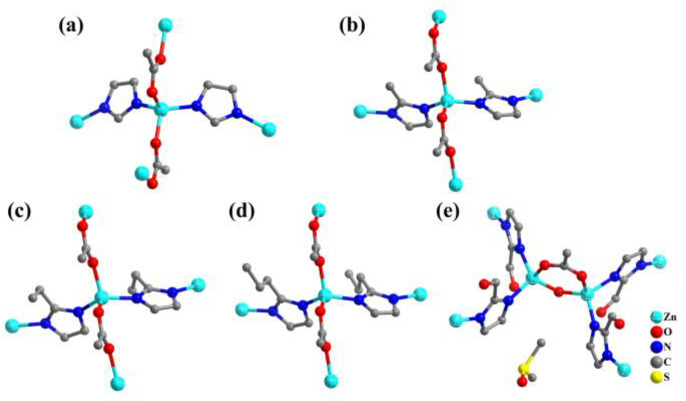
(**a**) The coordination environment of Zn atoms in TIF-A4. (**b**) The coordination environment of Zn atoms in TIF-A5. (**c**) The coordination environment of Zn atoms in TIF-A6. (**d**) The coordination environment of Zn atoms in TIF-A7. (**e**) The coordination environment of Zn atoms in TIF-A8.

In 2022, TIF-A4~A8 were synthesized in dimethyl sulfoxide with acetate auxiliary ligands and different imidazolate derivatives [66]. The acetate was from Zn(CH_3_CO_2_)_2_∙2H_2_O. TIF-A4 exhibited a 3D framework with dia topology, while TIF-A5~A8 had 2D layers with sql topology (Figure 5).

## 4. Special Properties of TIF-Ax

### 4.1. Solvent Stability

**Figure 6:**
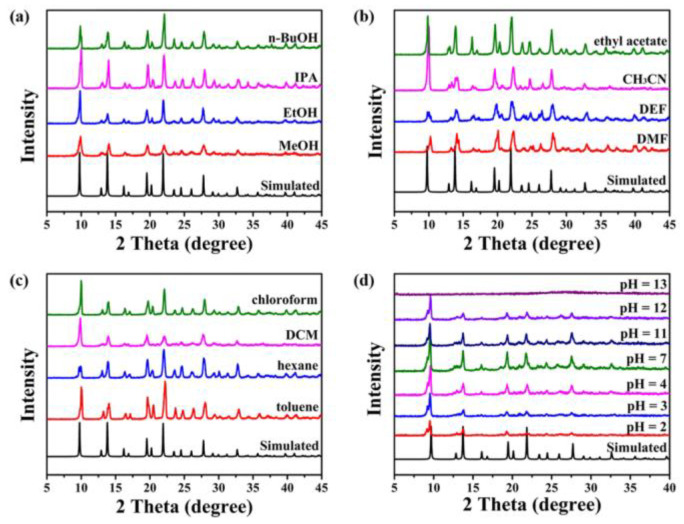
Powder XRD patterns of TIF-A1 under organic solvents (**a**–**c**) and different pH solutions (**d**) for 3 days. Adopted permission from Ref. [67]. Copyright © 2023, Elsevier Inc.

To realize the wide application of MOFs, they should first have good stability in harsh conditions, such as high temperature, high pressure, and acid and alkali environments. Interestingly, TIF-A1 can be stable in various organic solvents, such as water, *N*,*N*′-dimethylformamide (DMF), *N*,*N*′-diethylformamide (DEF), acetonitrile (CH_3_CN), ethyl acetate, toluene, n-hexane, dichloromethane (DCM), chloroform, methanol (MeOH), ethanol (EtOH), isopropanol (IPA), and n-butanol (n-BuOH). Furthermore, TIF-A1 can also maintain the framework after being soaked in a solution of pH= 2–11 (Figure 6) [67]. The exceptional stability of TIF-A1 makes it a promising candidate for further industrial applications.

### 4.2. Guest Selectivity

**Figure 7:**
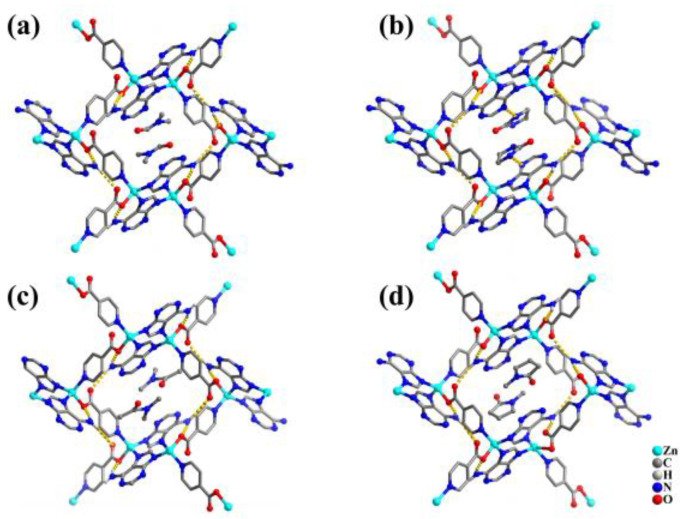
TIF-A1 with different guests: (**a**), DMF; (**b**), e-urea; (**c**), DMA; and (**d**), NMP.

**Scheme 3:**
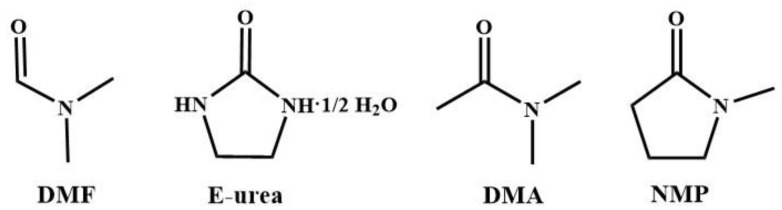
The solvents used for the synthesis of TIF-A1.

It is well known that solvent plays an important role in the structural variety of MOFs. Different solvent systems tend to produce different MOFs. However, it was found that the construction of TIF-A1 was independent of the solvents. TIF-A1 can accommodate amide solvents, including *N*,*N*′*-*dimethylformamide (DMF), ethyl urea (e-urea), *N*-methyl-1,2-pyrrolidone (NMP), and *N*,*N*′-dimethylacetamide (DMA) (Figure 7). Interestingly, the crystallization process of TIF-A1 exhibited a strict selective trapping ability for these four solvent guests, and its selective order was DMF > e-urea > NMP > DMA (Scheme 3, Table 3). This order of selection may be related to the size of the guest and the interaction between the host and guest. When the size of the guest is relatively small, the size of the guest is the main factor affecting the selection of guest molecules for TIF-A1. The four solvent guests are arranged in that order of molecular size: DMF < DMA < e-urea < NMP. DMF is very suitable to exist in the pores of TIF-A1. When the size of the guest is relatively large, TIF-A1 needs more energy to expand the pore to accommodate them. However, under this condition, the interaction between the host and the guest is dominant, and the energy needed to expand the pore will be partially offset. There is a strong N-H∙∙∙N hydrogen bond between e-urea and TIF-A1. There are non-classic C-H∙∙∙O and C-H∙∙∙N hydrogen bonds between NMP and TIF-A1. There is only a non-classic C-H∙∙∙O hydrogen bond between DMA and TIF-A1. Therefore, the selection order for TIF-A1 is e-urea > NMP > DMA. However, if the molecule is too large, it still depends mainly on the size of the guest. For example, TIF-A1 cannot trap 1,3-dimethyl-2-imidazolidinone (DMI) or *N*,*N*′-dimethylpropyleneurea (DMPU) [68].

### 4.3. Flexibility

**Figure 8:**
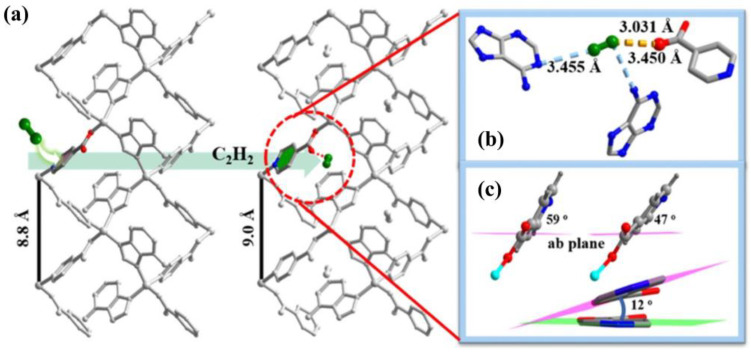
(**a**) Expansion of the c-axis of TIF-A1′ before and after C_2_H_2_ loading. (**b**) Strong interaction between C_2_H_2_ and int ligand of C_2_H_2_-loaded TIF-A1. (**c**) C_2_H_2_-induced rotation of int ligands. Adopted permission from Ref. [69]. Copyright © 2023, American Chemical Society.

As mentioned above, TIF-A1 showed a certain degree of contraction and expansion during crystallization to adapt to different guest molecules. Considering the porosity of TIF-A1, the flexibility of it was further explored by different gases [69]. The de-solvated form denoted as TIF-A1′ was obtained through methanol exchange. Unexpectedly, TIF-A1′ showed normal adsorption behavior for most common gases, such as N_2_, H_2_, and CO_2_, while it only showed multistep adsorption for C_2_H_2_. With the combination of in situ single-crystal XRD and calculation simulation, the flexibility of TIF-A1 was derived from the strong interaction between acetylene and the uncoordinated carboxylate O atoms of the int ligand. The C_2_H_2_-induced flexibility of TIF-A1 is intrinsic and was not affected by the sample size or defects. However, 2-NH_2_-TIF-A1 exhibited normal acetylene adsorption because the presence of a strong H-bond between the -NH_2_ of ad and the uncoordinated carboxylate O atoms of 2-NH_2_-int prevented C_2_H_2_ from approaching the active site and made the rotation of 2-NH_2_-int ligand more complex (Figure 8).

## 5. Application

### 5.1. CO_2_ Separation

Considering the presence of abundant active sites on TIF-A1, its CO_2_ absorption was first evaluated. After becoming fully activated, TIF-A1 exhibited high CO_2_ uptake at 273 K and 1 bar (107 cm^3^/g) [68]. In contrast, the highest CO_2_ adsorption in ZIFs at that time was ZIF-69, with a value of 70 cm^3^/g [22]. The high CO_2_ adsorption capacity of TIF-A1 can be attributed to the synergistic effect of int and ad ligands. Because the -NH_2_ group amino and pyrimidine N atoms in ad form an electron-rich system, they are potential CO_2_ bond sites. As mentioned above, the uncoordinated carboxylate O atoms of int ligand can also be active sites. In addition, the specific pore size and environment of TIF-A1 also play important roles in CO_2_ adsorption. Furthermore, the special interaction between TIF-A1 and CO_2_ indicates that TIF-A1 has the potential ability to selectively adsorb CO_2_, which makes it possible to apply TIF-A1 in the capture and separation of CO_2_ in flue gas. The flue gas mainly contains CO_2_ and N_2_, which are similar in molecular size, and it is difficult to achieve the separation effect by the pure physical adsorption of porous materials. The N_2_ adsorption of TIF-A1 was 4.7 cm^3^/g (273 K) and 1.4 cm^3^/g (298 K), which shows that TIF-A1 barely adsorbed N_2_. Henry’s constant indicates that the adsorption selectivity of TIF-A1 for CO_2_ over N_2_ was 90 (273 K,1 bar) and 60 (298 K,1 bar), respectively [62]. The excellent separation selectivity of TIF-A1 was further demonstrated by a breakthrough experiment [67].

### 5.2. NH_3_ Adsorption

**Figure 9:**
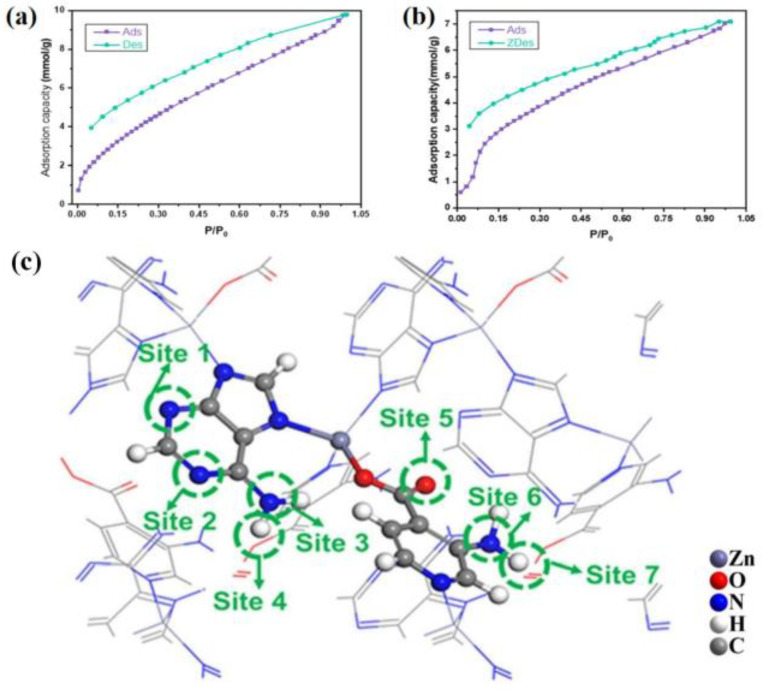
(**a**) NH_3_ adsorption–desorption isotherms of [Zn(3-NH_2_-int)(ad)](DMA). (**b**) NH_3_ adsorption–desorption isotherms of [Zn(3-NH_2_-int)(ad)](DMF). (**c**) NH_3_ adsorption sites in the frameworks of the fully activated [Zn(3-NH_2_-int)(ad)](DMA). Adopted permission from ref. [63]. Copyright © 2023, The Royal Society of Chemistry.

Due to the strong electronegativity of the N sites in TIF-A1, it can not only form an electron-rich system to attract CO_2_ but also form a strong hydrogen bond with NH_3_. Therefore, the NH_3_ adsorption of 3-NH_2_-TIF-A1 was studied. At 298 K and 1 bar, the maximum NH_3_ adsorption capacities of 3-NH_2_-TIF-A1 (obtained in DMA) was 9.8 mmol/g (Figure 9a, obtained in DMA) and 7.1 mmol/g (Figure 9b, obtained in DMF), which were higher than those of MOF-5 and ultrahigh porous MOF-177 [63]. 3-NH_2_-TIF-A1 has abundant active sites, including an uncoordinated oxygen atom and -NH_2_ group of 3-NH_2_-int, uncoordinated N atoms and -NH_2_ group of the ad ligand (Figure 9c). Among them, the -NH_2_ group on ad and the N of the pyrimidine ring at its para-position had lower *E*_ads_ values compared to those of the other four sites, which can provide more stable adsorption sites.

### 5.3. C_2_ Separation

**Figure 10:**
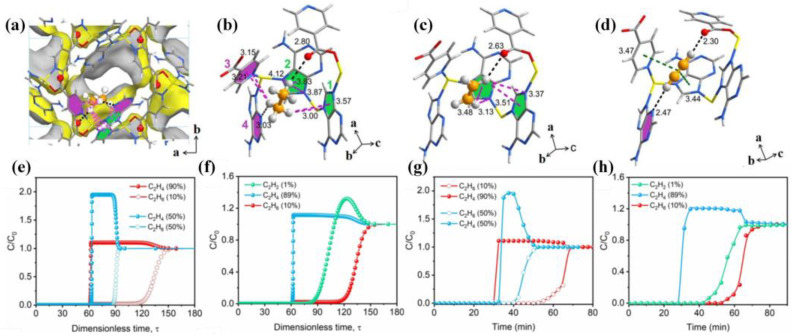
The refined structure of gas-loaded Zn(ad)(int). (**a**) Adsorption site of C_2_H_6_ in Zn(ad)(int) obtained from in situ PXRD analyses and structural refinements. (**b**–**d**) Local environment showing the binding modes of C_2_H_6_, C_2_H_4_, and C_2_H_2_ in Zn(ad)(int). Black, green, and pink dash lines refer to hydrogen bonding, π⋅⋅⋅π stacking, and C-H⋅⋅⋅π interaction, respectively. All interatomic distances are in angstroms. Simulated transient breakthrough curves and experimental dynamic breakthrough curves. (**e**,**f**) Simulated transient breakthrough curves for the separation of C_2_H_6_/C_2_H_4_ (10/90 and 50/50) and C_2_H_2_/C_2_H_6_/C_2_H_4_ (1/10/89) mixtures on Zn(ad)(int) at 298 K. (**g**,**h**) Experimental breakthrough curves for the separation of C_2_H_6_/C_2_H_4_ (10/90 and 50/50) and C_2_H_2_/C_2_H_6_/C_2_H_4_ (1/10/89) mixtures on Zn(ad)(int) at 298 K with a gas flow rate of 2 mL/min. Interatomic distances are in angstroms. Adopted permission from Ref. [70]. Copyright © 2023, Wiley-VCH GmbH.

In addition to CO_2_, TIF-A1 also exhibited a good C_2_ separation ability. In 2022, Ding and co-workers applied TIF-A1 to trap C_2_H_2_ and C_2_H_6_ simultaneously from the ternary mixture gas of C_2_H_2_/C_2_H_4_/C_2_H_6_ and performed the purification of C_2_H_4_ [70]. In the ternary mixture of C_2_H_2_/C_2_H_4_/C_2_H_6_, a strong electrostatic interaction occurred between C_2_H_2_ and the uncoordinated carboxyl O with high polarity in TIF-A1, and van der Waals (vdW) interaction occurred between C_2_H_6_ and the aromatic heterocycles with low polarity. TIF-A1 not only provides a strong binding site for the adsorption of C_2_H_2_ and C_2_H_6_, but also oblate C_2_H_6_ and linear C_2_H_2_ are very suitable for the spindle-shaped cage of TIF-A1, which can store the target molecule well (Figure 10a-d). The results show that TIF-A1 separated 99.9% ethylene from C_2_H_2_/C_2_H_4_/C_2_H_6_ (1/10/89) at 298 K and 1 bar, and the yield was 1.43 mmol/g, representing the best purification capacity at that time (Figure 10e–h).

### 5.4. CO_2_ Cycloaddition

In 2021, Wang and co-workers selected ZnX_2_ (X = Cl, Br, I) instead of ZnNO_3_ and selected a suitable solvent environment to synthesize a series of X-TIF-A1 with many halogen ions in the framework as catalysts for the CO_2_ activation reaction [71]. Due to their potent nucleophilic nature, halogen ions induced the ring-opening of propylene oxide, facilitating the reaction between CO_2_ and propylene oxide to generate propylene carbonate—a pivotal step in catalyzing CO_2_ conversion. The outcomes demonstrated that the yield of the CO_2_ activation reaction catalyzed by ZnI_2_-ad-int-DMF exhibited an upward trend with increasing reaction temperature. Notably, the yield achieved an impressive 98.5% at 140 °C. Subsequent to three cycles of reuse, ZnI_2_-ad-int-DMF experienced a moderate decline in catalytic activity. However, its framework remained stable despite exposure to high-temperature, high-pressure, and solvent conditions. Importantly, no discernible blocking phenomenon was observed as the iodine content decreased from 69 μmol/g to 51 μmol/g [71].

### 5.5. Heavy Metal Adsorption

**Figure 11:**
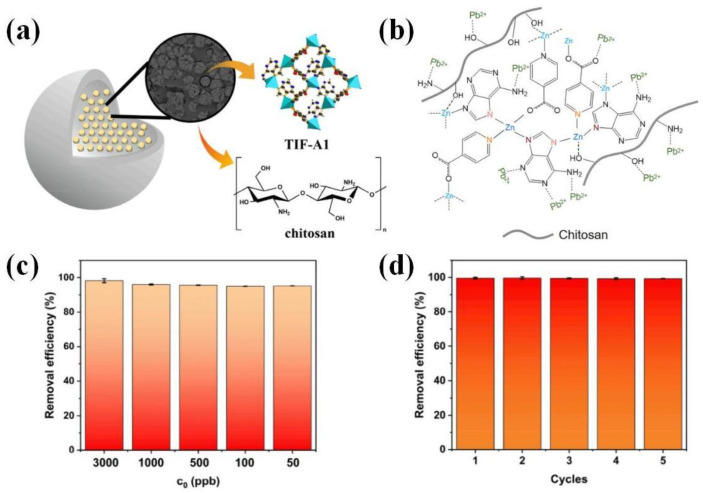
(**a**) Schematic depiction of the structure of TIF-A1/chitosan composite beads. (**b**) Schematic presentation of Pb(II) adsorption mechanism by TIF-A1/chitosan. (**c**) Removal efficiency of TIF-A1/chitosan for trace Pb(II) from reclaimed water. (**d**) Elution cycle experiment of TIF-A1/chitosan for Pb(II) (C_0_ = 0.1 mg/L). Adopted permission from Ref. [72]. Copyright © Elsevier B.V.

In 2022, Ma and co-workers used chitosan as the TIF-A1 growth template and prepared TIF-A1/chitosan composite beads using a secondary growth method for Pb(II) adsorption in water [72]. Several small TIF-A1 crystals formed rod-shaped clusters and aggregated in the pores of chitosan (Figure 11a). The average particle size of TIF-A1/chitosan was 0.2 cm. Such a large composite ball can effectively avoid the harm of pipeline blockage caused by the difficulty of separation and recovery of powdery MOF materials in practical applications. The polar groups -COO^−^, -OH, C=N, and -NH_2_ in the TIF-A1/chitosan structure coordinate with Pb(II) (Figure 11b).

The maximum adsorption of TIF-A1/chitosan for Pb(II) was 397.3 mg/g at 25 °C and pH = 6. Furthermore, in the mixed solution of multiple metal ions, TIF-A1/chitosan hardly adsorbed other ions, and the adsorption removal efficiency of Pb(II) was 99.17%. Especially when the concentration of Pb(II) was 100 ppb, the removal efficiency of trace Pb(II) was 99.95%, and the residual amount of Pb(II) met the international drinking water standards (Figure 11c) [72]. After five adsorption/desorption cycles, TIF-A1/chitosan could still maintain high adsorption performance, and the removal efficiency was more than 99% (Figure 11d). Furthermore, the crystal structure of TIF-A1 was not destroyed, and TIF-A1 was still attached to the chitosan matrix. In conclusion, TIF-A1/chitosan is a promising adsorbent for the removal of trace Pb(II) in drinking water treatment because of its excellent performance and reusability.

## 6. Conclusions and Outlook

In summary, TIF-Ax represents tetrahedral MOFs with auxiliary ligands, offering a promising avenue for diverse applications due to its versatile composition, intricate structure, and multifaceted functionalities. This paper provides an overview of TIF-Ax, encompassing its various structures, unique properties, synthetic methodologies, and potential applications. The progress achieved in the past decade is comprehensively summarized, highlighting the ample room for further advancements within the realm of TIF-Ax. While TIF-Ax exhibits great potential, there is room for enhancing its structural diversity and expanding its applications, especially when compared to well-established ZIFs. Within the realm of TIF-Ax’s structural diversity, the four components—metal centers, imidazole ligands, auxiliary ligands, and guest molecules—are all critical factors. Looking ahead, the development of novel TIF-Ax structures that exhibit both stability and enhanced functionality can be achieved by several means. This includes the introduction of a wider range of metal types or the combination of different metals, the exploration of innovative ligand combinations, and even the incorporation of chiral ligands. Moreover, the exploration of diverse synthetic systems holds promise for further advancements in this field.

### 6.1. Metal Center

In ZIFs, the commonly used bivalent cations with tetrahedral coordination are Zn^2+^ and Co^2+^ and Zn/Co bimetallic ZIFs. The introduction of Co ions can improve the light adsorption and light activity. In addition, Co^2+^ is magnetic and catalytic active, which gives multifunctionality to the final framework [73,74,75]. In terms of electrode preparation, Zn/Co-ZIF has been selected to enhance the conductivity of electrode materials through the synergistic effect of Zn/Co ions [76,77,78]. Thus, in the synthesis of TIF-Ax, it is possible to try to combine two central metals, Zn^2+^ and Co^2+^, or to combine other different tetrahedral central metals.

### 6.2. Ligands

There are two types of ligands in TIF-Ax—imidazole derivatives and auxiliary carboxylic acid ligand. On the one hand, the selection of ligands with functional groups is one of the effective methods to improve the performance of the framework, for example, reported imidazole ligands containing rich N sites (xanthine, hypoxanthine, 2,6-diaminopurine). Inspired by the synthesis of conductive MOFs, imidazole ligands containing an -SH group are also attractive ligands for electrically conductive metal-organic frameworks [79]. As for carboxylic acid ligands, other int ligands with functionalized groups include 2-hydroxyisonicotinic acid, 2,6-dichloroisonicotinic acid, and 2-fluoroisonicotinic acid, which are worth trying. For example, MOFs synthesized with carboxylic acid ligands containing an F group may be used in gas separation [80,81,82,83] and medical sterilization direction [84,85]. On the other hand, in addition to the functionalized modification methods above, chiral imidazole ligands or chiral carboxylic acid ligands can also be selected to construct chiral TIF-Ax for enantiomer resolution [86,87,88,89,90,91] and asymmetric catalysis [88,92,93,94]. For example, acetate can be replaced by natural amino acid chiral molecules.

### 6.3. Reaction System

In the synthesis of MOFs, the solvent has a great influence on the topology of the frame. During the reaction, the pressure generated by the solvent with the right boiling point will drive the reaction and the formation of pore channels, for which the appropriate volume of solvent molecules are selected to support the framework of MOFs through strong host–guest interaction. Formerly reported TIF-Ax have mainly been obtained in amide solvents, and the potential of new reaction systems, including DMSO [66,95], ionic liquids [96,97,98,99], surfactants [100,101,102], pyrazoles [103,104], and phenols [105,106], to synthesize TIF-Ax need to be explored.

In conclusion, TIF-Ax has emerged as significant porous material characterized by its excellent stability and multifunctionality and underscored by its potential for mass production and versatile applications across various fields. Furthermore, the synthesis of TIF-Ax allows for the flexible combination of different components, thereby offering a wide array of choices in component selection. This aspect not only serves as a wellspring of inspiration but also presents substantial room for subsequent innovations in the design and evolution of TIF-Ax materials.

The exceptional properties of TIF-Ax, including its structural adaptability, robust stability, and diverse functionalities, position it as a material of great promise. Its synthesis methodology, which encourages creative amalgamation of various components, opens avenues for the discovery of novel combinations that could yield even more remarkable properties. As researchers delve deeper into the realm of TIF-Ax, the horizon of possibilities expands, beckoning further exploration, innovation, and transformative applications.

## Figures and Tables

**Table 1:** Summary of reported TIF-Ax.

Name	Formula	Space Group	Topology	Ref.
TIF-A1	[Zn(ad)(int)](DMF) ^1^	*Pna*2_1_	dmp	[22]
TIF-A2	Zn_2_(im)_3_(int) ^2^	*Pca*2_1_	dia	[22]
TIF-A3	Zn_2_(im)(int)_2_(OH)	*C*2*/c*	neb	[22]
2-NH_2_-TIF-A1	[Zn(ad)(2-NH_2_-int)](DMF)	*Pna*2_1_	dmp	[62]
3-NH_2_-TIF-A1	[Zn(3-NH_2_-int)(ad)](DMF) [Zn(3-NH_2_-int)(ad)](DMA) ^3^	*Pna*2_1_	dmp	[63]
Zn-thp-nit	[Zn(thp)(nit)] ^4^	*Pbca*	—	[64]
Cd-ad-int	[Cd_2_(ad)_2_(int)_2_(DMF) (H_2_O)](DMF)	*P*2_1_*/n*	mog	[65]
TIF-A4	Zn(im)(Ac) ^5^	*Ima*2	dia	[66]
TIF-A5	Zn(2-mim)(Ac) ^6^	*P*2_1_*/c*	sql	[66]
TIF-A6	Zn(2-eim)(Ac) ^7^	*P*2_1_*/c*	sql	[66]
TIF-A7	Zn(2-pim)(Ac) ^8^	*Pna*2_1_	sql	[66]
TIF-A8	[Zn_2_(OH^-^)(Ac)(2-cim)_2_](DMSO) ^9^	*Cmc*2_1_	sql	[66]

^1^ ad = adeninate; int = isonicotinate; DMF = *N*,*N*′-dimethylformamide; ^2^ im = imidazolate; ^3^ DMA = *N*,*N*′-dimethylacetamide; ^4^ thp = theophylline; nit = nicotinic acid; ^5^ Ac = acetic acid; ^6^ mim = 2-methylimidazolate; ^7^ eim = 2-ethylimidazolate; ^8^ pim = 2-propylimidazolate; ^9^ cim = imidazolate-2-carboxaldehyde; DMSO = dimethyl sulfoxide.

**Table 2:** Data comparison of TIF-A1 synthesized by different methods.

Synthesis Method	T (℃)	Time	BET (m^2^/g)	Yield (%)
Room temperature	r.t.	3 days	470.27	14
Room temperature	r.t.	2.5 h	263.10	30
Ultrasonic	r.t.	2.5 h	107.69	31
Reflux (ZnO)	120	30 min	535.79	75
Reflux (Zn(CH_3_COO)_2_∙2H_2_O)	120	30 min	536.38	80
Reflux (Zn(NO_3_)_2_∙6H_2_O)	120	30 min	549.97	83
Upscale synthesis	120	8 h	490.66	62
Original method	120	3 days	681.08	88

**Table 3:** The statistics of the guest selection in different solvents.

Solvents	Product
DMF	TIF-A1⋅DMF
DMF/formamide	TIF-A1⋅DMF
DMF/e-urea	TIF-A1⋅DMF
DMF/DMA	TIF-A1⋅DMF
DMF/NMP	TIF-A1⋅DMF
DMF/DMA/NMP/e-urea	TIF-A1⋅DMF
E-urea	TIF-A1⋅e-urea
E-urea/DMA	TIF-A1⋅e-urea
E-urea/NMP	TIF-A1⋅e-urea
E-urea/DMA/NMP	TIF-A1⋅e-urea
NMP/DMA/dmurea/MeOH	TIF-A1⋅NMP
NMP/dmurea/MeOH	TIF-A1⋅NMP
DMA/dmurea/MeOH	TIF-A1⋅DMA
DMA/NMP	None
DMI	None
DMPU	None
Formamide	None
Urea	None
Dmurea	None

## Data Availability

Not applicable.
